# Infections in Infancy and Association with Long-Term Weight Gain and Obesity in Children, Southern California, USA

**DOI:** 10.3201/eid3208.241522

**Published:** 2026-08

**Authors:** Zackary W. Taylor, Jane C. Lin

**Affiliations:** Kaiser Permanente Southern California at KP Los Angeles Medical Center, Los Angeles, California, USA (Z.W. Taylor); Kaiser Permanente, Pasadena, California, USA (J.C. Lin)

**Keywords:** obesity, viruses, viral infections, respiratory infections, antimicrobial drugs, weight gain, children, California, United States

## Abstract

Chronic obesity is a growing public health threat in the United States; >40% of adults are affected. We retrospectively assessed the frequency of medically attended infection in infants born during 2008–2012 relative to absolute weight and body mass index outcomes at age 4–6 years of age and 10–15 years of age. Multivariable regression revealed those with early clinical infection had higher rates of obesity at 4–6 years and 10–15 years than those without, proportional to the number of infections diagnosed and corresponding to significant weight increases. Compared with infants whose infections were not treated with antimicrobial drugs, treatment was less predictive of weight outcomes. Most infections were viral. Our investigation found a correlation between infections diagnosed in infancy and later weight outcomes, but we could not determine the cause.

Obesity is a major public health threat in the United States; >40% adults are affected, and obesity is a risk factor for chronic diseases such as diabetes and heart disease (*1*). Obesity itself is also recognized as a chronic disease that has a cumulative relation to mortality that is especially marked when starting earlier in life (*2*–*4*) or prolonged over a decade (*5*). Childhood obesity is a growing problem; ≈1 in 5 children and adolescents are now obese (*6*), and adolescent obesity frequently leads to adult obesity. Chronic obesity, although traditionally thought of as a condition of choice, is socioeconomically, genetically, and biologically driven, with complicated and multifactorial roots emphasized in US medical practice guidelines (*7*). Infections are well-established drivers of other chronic diseases, and a well-recognized link between infections and long-term weight loss (e.g., tuberculosis, parasites) belies a less self-explanatory link between early viral infection and long-term weight gain and obesity, observed in the United States and globally.

This link between infection and long-term weight gain was observed in a longitudinal prospective study of in the Philippines (*8*) observed from infancy. The participants in that study were followed under the hypothesis that early infections and other adverse conditions in infancy and early childhood would be correlated with long-term decreased weight relative to uninfected patients, but a correlation of prospectively reported early upper respiratory infections with long-term increase in body mass index (BMI) as adults was observed. Infection-related antimicrobial drug exposure was a possible explanation for the increased BMI finding. The study did not control for antimicrobial drug use, and antimicrobial drugs have been associated with weight gain and obesity (*9*–*12*). However, a subsequent large retrospective study in the United States revealed early antimicrobial drug exposure to be a proxy for early infection and association with obesity, rather than the opposite. Medically attended infections in infancy, defined as infections for which medical care was sought, were shown to be independent predictors of obesity, and the risk of obesity increased in proportion to the number of diagnosed infections (*13*). Within that study, the correlation with medically attended infections in infancy and long-term obesity was also confirmed in a subset of same-sex twins with discordant infection and antimicrobial drug exposure status, corroborating the observed correlation by nullifying parental care-seeking behavior and other parental confounders. The correlation of early antimicrobial drugs and obesity in humans, observed previously in many retrospective studies, might not have been observed if previous studies had controlled for the underlying infections; any conclusion that antimicrobial drug use in infancy leads to obesity could be erroneous.

The use of obesity as the outcome variable in those retrospective studies has also been questioned. A change in the rate of obesity, rather than absolute weight, is usually used to depict clinically notable weight change in a population, because obesity is associated with meaningful health outcomes. However, obesity diagnosis is made on the basis of a BMI threshold, which has been discounted as a possible self-confounder in regression analysis (*14*), favoring absolute weight velocity as an alternative to BMI. In our population of children at Kaiser Permanente Southern California Kaiser (KPSCAL), we sought to confirm the previously observed association of childhood infections in infancy with weight increase and obesity later in childhood, to analyze the magnitude of the absolute weight increase, and to find whether absolute weight differences are commensurate with obesity rate differences. Antimicrobial drug use is a possible confounder for infection, including viral infections during which antimicrobial drugs are often prescribed, so antimicrobial drug–treated infections were assessed separately.

## Methods

### Population and Study Design

**Figure 1:**
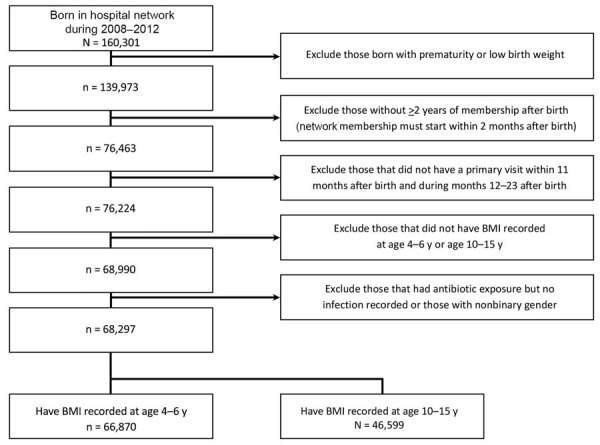
Flowchart for inclusion or exclusion of children in retrospective study of infant infections and obesity in children, southern California, USA. BMI, body mass index.

Our study identified a retrospective, longitudinal cohort of infants born 2008–2012 in the KPSCAL system. Among that birth cohort, we excluded patients with low birthweight (weight <2,500 g), prematurity (gestational age <37 weeks), without KPSCAL membership for the first 2 years of life, without primary care visits for each of the first 2 years, with missing outcome measurements at 4–6 years of age and at 10–15 years of age, those with nonbinary gender identities, or with antimicrobial drug exposure without a concurrent infection diagnosis (Figure 1).

Outcome measures included obesity status, (i.e., BMI >95%) and absolute weight (kg), as measured at age 4–6 years of age, 10–15 years of age, or both. We used BMI and absolute weight closest to age 5 for those 4–6 years of age and age 10 for those 10–15 years of age. We standardized BMI by the age and sex of the child on the basis of Centers of Disease Control and Prevention growth charts by using the associated macro (https://www.cdc.gov/growth-chart-training/hcp/computer-programs). We used absolute weight rather than standardized weight (z-score) specifically because absolute weight differences are more readily understood and communicated in terms of clinical importance. We accounted for weight differences related to sex and height by including those variables in multivariable analysis and accounted for age by separation into 2 discrete age groups.

We divided patients into 3 groups: those with no medically attended infections in the first year of life (NMAI); those with medically attended infections without antimicrobial drug treatment (IWOT); and those with *>*1 medically attended infections with antimicrobial drug treatment (IWAT). Antimicrobial drug use was thereby removed as a possible confounder in the NMAI versus IWOT comparison, enabling us to gauge the association of infection with risk for weight gain and obesity, independent of antimicrobial drugs. We compared the IWOT group to the IWAT group to determine any additional contribution of antimicrobial drugs to weight outcomes. We also assessed the frequency of medically attended infection in infancy (<12 months of age), timing of first infection (<6 vs. 6–12 months of age), antimicrobial drug class (narrow spectrum vs. broad spectrum), frequency (number of courses), and timing of first antimicrobial drug (<6 vs. 6–12 months of age). We classified the use of antimicrobial drugs in infancy in >1 instance in the IWAT group and recorded the number of infections in that group separately from the number of antimicrobial drug courses. We considered infections recorded >2 weeks apart separate infections, and if there were multiple infection diagnoses within a single 2-week period, even with differing International Classification of Diseases, 9th Revision, codes, they were counted as a single clinical infection. We defined narrow-spectrum antimicrobial drug exposure as exposure to only amoxicillin, penicillin, a first-generation cephalosporin, or any combination of those. We defined broad-spectrum antimicrobial drug exposure as exposure to any other antimicrobial drug or combination in infancy (<12 months of age), whether or not the patient also received a narrow-spectrum antimicrobial drug.

The KPSCAL healthcare system is a large, comprehensive healthcare model, with clinics and hospitals throughout Southern California providing inpatient and outpatient care, incorporating primary and subspecialty care to minimize the need to seek care outside the system. Although care might not be captured on the KPSCAL electronic medical record (EMR) if patients get care while traveling outside southern California, and some KPSCAL patients do get contracted outside care for certain highly specialized procedures (e.g., transplants during the acute transplant period, pediatric cardiothoracic surgery), member patients otherwise obtain healthcare within KPSCAL, including urgent care, emergency visits, and hospitalizations. This system enables reliable tracking and analysis of nearly all medically attended infections among active Kaiser members. We extracted data from the KPSCAL EMR; research was funded by a grant from the Southern California Regional Research Committee and was conducted under the approval of the KPSCAL institutional review board.

### Power Analysis, Covariates and Statistical Analysis

The known prevalence of obesity in the pediatric population is ≈20%, and a power analysis revealed that an 80% power to detect a significant difference of 1% in overall obesity (5% increase in obesity rate) between any 2 groups would require 25,880 population in each group. A 5-year birth cohort at KPSCAL is ≈150,000 patients and, assuming 50% attrition of the birth cohort within the first 2 years, would yield a study population of 75,000. That population would enable the possibility of obtaining the threshold 5% difference in obesity between >2 of the 3 groups.

Additional covariates included in the study were maternal obesity (BMI <30 vs. >30); macrosomia (>4,000 g birthweight); delivery type (cesarean section vs. vaginal); breastfeeding (defined as nonbreastfed, partially breastfed, or exclusively breastfed at 4 months of age); sex, race and ethnicity; and estimated household income. We included those covariates because each is known to be correlated with childhood obesity, and in the case of sex, breastfeeding status, race and ethnicity, and household income are also known to be correlated with infection diagnoses. Race and ethnicity included Asian or Pacific Islander, Black, Hispanic, White, and other or unknown.

We evaluated baseline characteristics by study groups by using χ^2^ test for categorical variables and Kruskal-Wallis test for continuous variables. We compared obesity and absolute weight outcomes in NMAI versus IWOT, and IWOT versus IWAT, by using logistic regression analysis for obesity outcomes and linear regression for absolute weight. Covariates included maternal obesity, macrosomia, breastfeeding, delivery type, sex, and race and ethnicity when comparing NMAI versus IWOT or IWAT. We further adjusted the number of infections when comparing IWOT versus IWAT. We included height in the model when assessing relationship between study groups and absolute weight. We prescreened the dataset for improbable height and weight values for age and excluded visits containing such values from the analysis. We checked multicollinearity with variance inflation factor with no results >5, suggesting multicollinearity was not an issue. Statistical significance was set at p<0.05. We conducted analyses by using R statistical software version 4.3.0 (The R Project for Statistical Computing, https://www.r-project.org).

We did not differentiate the type and severity of infections diagnosed in our statistical analysis, but after the analysis was complete, we assessed the most common diagnostic codes in each group. We identified the most common individual infection codes from the International Classification of Diseases, 9th Revision, and the most common categories of infection. We considered the following categories: viral infections (e.g., acute nasopharyngitis and specific viral diagnoses such as respiratory syncytial virus or rotavirus), viral-associated infections (which might be bacterial but are often accompanied or preceded by a virus, such as otitis media and pneumonia), and nonviral infections (infections that are generally not caused by, accompanied by, or preceded by viral illness).

In addition, we approximated the population attributable fraction (PAF) to give an estimate of the exposure effect to medically attended infections to obesity in our population, by using the odds ratio (OR) from our primary multivariable analysis for the 10–15 years of age group. On the basis of primary analysis, which showed no additional contribution of antimicrobial drug use to obesity outcomes, the 2 populations of IWOT and IWAT were combined for the PAF analysis to create the total population exposed to medically attended infections by using the following calculation: PAF = P_e_ (OR − 1)/(P_e_ [OR −1] + 1), where P_e_ is the proportion of patients exposed to clinical infection, determined by the equation (IWOT+IWAT)/(IWOT+IWAT+NMAI) (Table 1), and OR is from the outcome of the multivariate analysis for IWOT versus NMAI in that age group (Table 2). A range of PAF can be calculated by using the endpoints of the OR 95% CI in the calculation, recalculating for each endpoint.

**Table 1:** Maternal and infant characteristics, infant exposures, and weight and obesity outcomes at 10–15 years of age from a study of the effect of infant infections on obesity later in life*

Characteristics	NMAI, n = 7,594	IWOT, n = 18,468	IWAT, n = 20,537	p value
Maternal factors				
Maternal obesity	620 (8.2)	1,811 (9.8)	2,341 (11)	<0.001
Caesarean section	2,356 (31)	5,646 (31)	6,551 (32)	0.017
Household income†				<0.001
$0–30,000	306 (4.0)	932 (5.0)	1,060 (5.2)	
$30,001–50,000	1,722 (23)	4,622 (25)	5,404 (26)	
$50,001–70,000	2,103 (28)	5,473 (30)	6,063 (30)	
$70,001–90,000	1,674 (22)	3,824 (21)	4,174 (20)	
>$90,000	1,789 (24)	3,617 (20)	3,836 (19)	
Race/ethnicity				<0.001
Asian/Pacific Islander	1,520 (20)	2,638 (14)	2,363 (12)	
Black	491 (6.5)	1,426 (7.7)	1,527 (7.4)	
Hispanic	3,002 (40)	9,217 (50)	10,968 (53)	
White	2,401 (32)	4,852 (26)	5,357 (26)	
Other/unknown	180 (2.4)	335 (1.8)	322 (1.6)	
Infant factors				
Sex				
F	4,071 (54)	9,327 (51)	9,337 (45)	<0.001
M				
Macrosomia, birthweight >4 kg	693 (9.1)	1,781 (9.6)	2,376 (12)	<0.001
Age at first antibiotic exposure, mo				
<6			8,339 (41)	
6–12			12,198 (59)	
No. antibiotic courses by age 12 mo				
0	7,594 (100)	18,468 (100)	NA	
1	NA	NA	12,523 (61)	
2	NA	NA	5,292 (26)	
>3	NA	NA	2,722 (13)	
No. clinical infections by age 12 mo				<0.001
0	7,594 (100)	0 (0)	0 (0)	
1	NA	8,690 (47)	3,429 (17)	
2	NA	5,598 (30)	5,545 (27)	
3	NA	2,749 (15)	5,173 (25)	
>4	NA	1,431 (7.7)	6,390 (31)	
Breastfeeding status				<0.001
Nonexclusive	2,148 (28)	5,257 (28)	5,802 (28)	
Exclusive	2,619 (34)	5,307 (29)	5,259 (26)	
Nonbreastfed	2,642 (35)	7,413 (40)	8,958 (44)	
Unknown	185 (2.4)	491 (2.7)	518 (2.5)	
Outcomes at 10–15 years of age				
Age, y, median (IQR)	10.0 (10.0–11.0)	10.0 (10.0–11.0)	10.0 (10.0–10.0)	<0.001
Height, cm, median (IQR)	145.0 (139.7–151.1)	145.0 (139.7–151.3)	145.0 (139.7–151.1)	0.5
Weight, kg, median (IQR)	39.6 (33.0–49.2)	41.0 (33.8–50.7)	41.5 (34.2–52.0)	<0.001
Obesity	1,485 (20)	4,384 (24)	5,564 (27)	<0.001
BMI subcategories				<0.001
Normal, 5th–85th percentile	4,523 (60)	10,073 (55)	10,682 (52)	
Underweight, <5 percentile	332 (4.4)	674 (3.6)	585 (2.8)	
Overweight, 85–95 percentile	1,254 (17)	3,337 (18)	3,706 (18)	
Class I obesity‡	983 (13)	2,833 (15)	3,527 (17)	
Class II obesity‡	364 (4.8)	1,124 (6.1)	1,456 (7.1)	
Class III obesity‡	138 (1.8)	427 (2.3)	581 (2.8)	

*Values no. (%) except as indicated. IWAT, clinical infection in infancy with antimicrobial drug treatment; IWOT, clinical infection in infancy without antimicrobial drug treatment; NA, not applicable; NMAI, no clinical infections in infancy. †Income bracket estimated by the mean household income for area of residence ‡Standardized for age and gender. Childhood obesity categories class I, BMI >95%–<120% of 95 percentile; class II, BMI >120%–<140% of 95 percentile; class III, >140% of 95 percentile.

**Table 2:** Multivariable analysis of IWOT versus NMAI by age, obesity, and weight gain in children from California, USA*

Characteristic	Odds ratio (95% CI)	p value
Age 4–6 y		
IWOT–obesity association		
IWOT vs. NMAI	1.09 (1.02–1.18)	0.02
No. clinical infections		
1	1.04 (0.95–1.13)	0.4
2	1.11 (1.01–1.22)	0.03
3	1.19 (1.06–1.33)	0.004
>3	1.2 (1.04–1.39)	0.013
Age at first clinical infection		
<6 mo	1.1 (1–1.21)	0.05
>6 mo	1.02 (0.92–1.11)	0.8

IWOT-associated weight gain, kg		
IWOT vs. NMAI	0.09 (0.04–0.14)	0.001
No. clinical infections		
1	0.04 (−0.02 to 0.1)	0.2
2	0.14 (0.07–0.21)	<0.001
3	0.11 (0.01–0.2)	0.03
>3	0.19 (0.06–0.32)	0.004
Age at first clinical infection, mo		
<6	0.08 (0.01–0.15)	0.02
>6	0.02 (−0.04 to 0.09)	0.5

Age 10–15 y		
IWOT–obesity association		
IWOT vs. NMAI	1.11 (1.04–1.19)	0.003
No. clinical infections		
1	1.06 (0.97–1.14)	0.2
2	1.11 (1.01–1.21)	0.02
3	1.2 (1.07–1.33)	0.001
>3	1.3 (1.13–1.49)	<0.001
Age at first clinical infection, mo		
<6	1.04 (0.95–1.15)	0.4
>6	1.1 (1.01–1.2)	0.02

IWOT-associated weight gain, kg		
IWOT vs. NMAI	0.46 (0.21–0.71)	<0.001
No. clinical infections		
1	0.21 (−0.08 to 0.49)	0.2
2	0.52 (0.16–0.82)	0.004
3	0.88 (0.45–1.31)	<0.001
>3	1.19 (0.62–1.76)	<0.001
Age at first clinical infection, mo		
<6	0.23 (−0.11 to 0.57)	0.2
>6	0.38 (0.07–0.68)	0.02

*IWOT, clinical infection in infancy without antimicrobial drug treatment; NMAI, no clinical infections in infancy.

## Results

**Figure 2:**
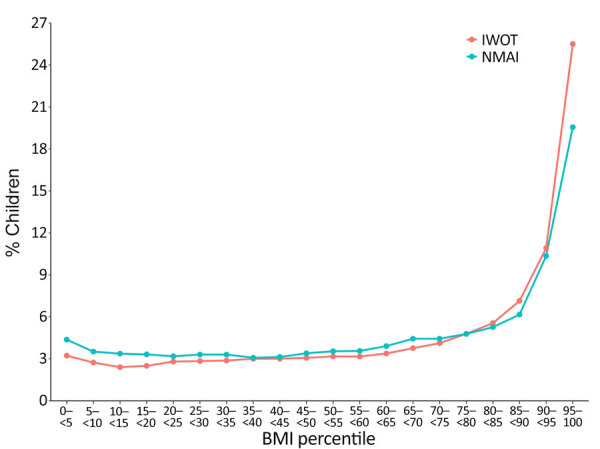
Population proportion among children 10–15 years of age by BMI percentile and infant clinical infection status from study of infant infections and obesity in children, southern California, USA. We used BMI and absolute weight closest to age 10 for those 10–15 years of age. BMI, body mass index; IWOT, clinical infection in infancy without antimicrobial drug treatment; NMAI, no clinical infection in infancy.

We identified 66,870 children at 4–6 years of age and 46,599 at 10–15 years of age for final analysis (Figure 1). Of the 10–15 years of age group, 7,594 had NMAI, 18,468 had IWOT, and 20,537 had IWAT, and antimicrobial drug treated patients were more likely to have had multiple clinical infections (53% of IWOT group vs. 83% of IWAT group had >1 infection diagnosed) (Table 1). Of the 10–15 years of age group, 98.7% (n = 45,981) of patients had weights extracted at 10–12 years of age and the rest at 13–14 years of age (including 85 patients at 14 years of age). Children with NMAI became obese at a rate of 9.7% at 4–6 years of age and 20% by 10 years of age, whereas the IWOT group had higher rates of obesity (12% by 4–6 years of age, 24% by 10 years of age). On univariate analysis, the increase in obesity was distributed among all obesity classes, from mild to severe (Table 1; Figure 2).

**Figure 3:**
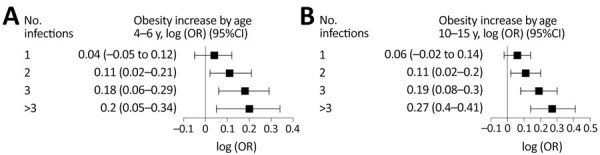
Increase in later obesity by number of clinical infections in infancy from study of infant infections and obesity in children, southern California, USA. A) Obesity by 4–6 years of age. B) Obesity by 10–15 years of age. OR, odds ratio.

**Figure 4:**
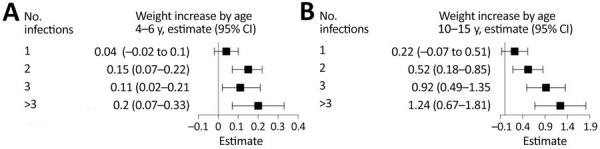
Increase in later weight (kilograms) by number of clinical infections in infancy from study of infant infections and obesity in children, southern California, USA. A) Weight increases by 4–6 years of age. B) Weight increases by 10–15 years of age.

The increase in odds of obesity in IWOT versus NMAI was significant in multivariable analysis at 4–6 years of age (OR 1.09, 95% CI 1.02–1.18; p = 0.02) and 10–15 years of age (OR 1.11, 95% CI 1.04–1.19; p = 0.003) and increased in proportion to the number of clinical infections (Tables 2, 3; Figure 3). Median weight in the NMAI group was 18.6 kg at 4–6 years of age and 39.6 kg at 10–15 years of age and slightly higher in IWOT (19.0 kg at 4–6 years, 41 kg at 10 years). The weight increase was small but significant in multivariable analysis at 4–6 years of age (0.09 kg, 95% CI 0.04–0.14 kg; p = 0.001); and at 10–15 years of age (0.46 kg, 95% CI 0.21–0.71 kg; p<0.001) and was proportional to the cumulative number of clinical infections (Tables 2, 3; Figure 4). We found no clear relationship in the timing of first infection (<6 vs. >6 months of age) with later weight gain or odds of obesity. In contrast to the persistent correlation between medically attended or untreated infections and subsequent obesity, we found no difference in the subsequent risk for obesity in the IWAT group compared with IWOT in multivariable analysis by age 10–15 years of age (obesity at 10–15 years in IWAT vs. IWOT, OR 1.05, 95% CI 0.99–1.1; p = 0.08). There was a small increase in absolute weight at 10–15 years of age in the antimicrobial treated group (vs. IWOT; 0.23 kg, 95% CI 0.02–0.44 kg; p = 0.03), but that difference did not appear to be dose-dependent (Tables 2, 3). In subgroup analysis, antimicrobial drug use did show a significant correlation with later obesity when broad-spectrum agents were used (vs. IWOT: at 4–6 years, OR 1.09, 95% CI 1.01–1.17; p = 0.02; at 10–15 years, OR 1.09, 95% CI 1.02–1.17; p = 0.01) (Tables 2, 3) and with absolute weight increase when broad-spectrum agents were used or when there was very early exposure (at <6 months of age) to antimicrobial drugs (Tables 2, 3).

**Table 3:** Multivariable analysis of IWAT versus IWOT by age, obesity, and weight gain in children from California, USA*

Characteristics	Odds ratio (95% CI)	p value
Age 4–6 y		
Antimicrobial drug-obesity association		
IWAT vs. IWOT	1.06 (1–1.12)	0.05
No. courses		
1	1.05 (0.99–1.12)	0.1
2	1.1 (1.01–1.2)	0.02
>2	1 (0.89–1.12)	0.9
Antimicrobial drug class		
Narrow-spectrum	1.04 (0.98–1.11)	0.2
Broad-spectrum	1.09 (1.01–1.17)	0.02
Age at first antimicrobial drug, mo		
<6	1.11 (1.03–1.19)	0.006
>6	1.03 (0.97–1.09)	0.4
Antimicrobial drug–associated weight gain, kg		
IWAT vs. IWOT	0.06 (0.01–0.11)	0.01
No. courses		
1	0.05 (0–0.1)	0.04
2	0.09 (0.02–0.17)	0.02
>2	0.05 (−0.06–0.15)	0.4
Antibiotic class		
Narrow	0.05 (0–0.1)	0.06
Broad spectrum	0.08 (0.02–0.14)	0.01
Age at first antimicrobial drug, mo		
<6	0.11 (0.04–0.17)	0.001
>6	0.03 (−0.02–0.08)	0.2
Age 10–15 y		
Antimicrobial drug–obesity association		
IWAT vs. IWOT	1.05 (0.99 - 1.1)	0.08
No. courses		
1	1.04 (0.98 – 1.1)	0.2
2	1.1 (1.02 – 1.19)	0.02
>2	1.01 (0.9 - 1.12)	0.9
Antimicrobial drug class		
Narrow-spectrum	1.02 (0.96–1.08)	0.5
Broad spectrum	1.09 (1.02–1.17)	0.01
Age at first antimicrobial drug, mo		
<6	1.06 (1–1.14)	0.07
>6	1.04 (0.98–1.12)	0.2
Antimicrobial drug–associated weight gain, kg		
IWAT vs. IWOT	0.23 (0.02–0.44)	0.03
No. courses		
1	0.19 (−0.04–0.41)	0.1
2	0.39 (0.06–0.72)	0.02
>2	0.18 (−0.27–0.64)	0.4
Antimicrobial drug class		
Narrow-spectrum	0.13 (−0.11–0.37)	0.3
Broad spectrum	0.4 (0.13–0.67)	0.004
Age at first antimicrobial drug, mo		
<6	0.32 (0.05–0.6)	0.02
>6	0.18 (−0.06–0.41)	0.1

*IWAT, clinical infection in infancy with antimicrobial drug treatment; IWOT, clinical infection in infancy without antimicrobial drug treatment.

Most clinical infections were viral, and most of the patients with infections had *>*1 medically attended upper respiratory infection. In both IWOT and IWAT groups, the most common single infection diagnosis was acute upper respiratory infection of unspecific site (International Classification of Diseases, 9th Revision, code 465.9), diagnosed 17,179 times in the IWOT group and 20,081 times in the IWAT group. Most documented infections were either viral (e.g., upper respiratory infections or bronchiolitis) or viral-associated (e.g., otitis media); a total of 43,306 viral or viral-associated diagnoses occurred in patients with IWOT and 73,181 in the IWAT group, including 19,973 diagnoses of otitis media. Nonviral infections with a known pathogen were far less common; urinary tract infection was the most frequent (941 documented urinary tract infections in the IWAT group), and oral candidiasis was the most common nonviral diagnosis overall in both infected groups (2,200 cases in the IWOT group, 1,986 cases in the IWAT group).

We calculated the PAF for obesity at 10–15 years of age by using OR 1.11 (95% CI 1.04–1.19), yielding a risk of 8.4% (95% CI 3.2%–13.7%) for obesity attributable to early clinical infections. However, the use of OR rather than relative risk for a nonrare outcome (i.e., >10% risk) in calculating PAF will falsely elevate the attributable risk, albeit probably by <25% for an outcome affecting 20% of the population (*15*). Thus, a more conservative estimate of obesity attributable to early clinical infections would be ≈2.4%–10%.

## Discussion

Our study confirms the link between infections in infancy and obesity later in life. We found a significant increase in odds of obesity at later ages for those with infant infections and increasing risk proportional to the number of infections diagnosed. This increasing proportional risk with cumulative exposure to infections makes a biological effect more likely, whereas the lack of persistent correlation between antimicrobial drug use and obesity and lack of dose-dependence with antimicrobial drugs and obesity in our study make the effect of antimicrobial drugs on obesity appear less predictable. The later weight gain related to IWOT (0.09 kg by 4–6 years of age, 0.46 kg by 10–15 years of age) could be dismissed as clinically insignificant were it not for the accompanying significant increase in obesity. A similar small weight gain was dismissed in another study (*14*), despite a significant associated increase in obesity rate, but such a dismissal of obesity rate increases accompanied by only small absolute weight gains in a population might inappropriately discount a clinically highlighted weight difference limited to a subpopulation. Subpopulations already prone to obesity (e.g., patients already overweight or obese) will gain weight at a more rapid rate with social or environmental perturbations than most children (*16*). Moreover, the United States has increasing rates of obese children at 21%, with another 15% overweight (*17*), and a small weight gain restricted to a population on the verge of obesity might push a large number in this skewed distribution into the category of obesity. Perhaps 2.4%–10% of the obesity risk in our population is attributable to early infections on the basis of PAF analysis. If applied to a large population such as the pediatric population of the United States, even such a small, heterogeneous increase in obesity could represent hundreds of thousands of children. In the United States, where obesity is considered epidemic and is a well-recognized public health issue, identifying and addressing even a risk of relatively small magnitude could begin to stem the rising tide of obesity. 

Our mathematical attribution of risk is limited, however, as we cannot attribute causality to this observed link between medically attended infection and obesity. Our study is dependent on data available from the EMR, and our population was drawn from a relatively limited locale (southern California, USA). Southern California has a large Hispanic population (Table 1), which is more prone to obesity and is not representative of the United States population at large. Moreover, care-seeking behavior might differ between populations. Although the Hispanic population in particular is not known to seek care at higher rates and differences in care-seeking behaviors between large racial and ethnic subgroups would have been controlled for in our multivariable analysis, it is possible there are unidentified subgroups who are more prone to both obesity and care-seeking for their infants. Such confounding could lead to an observed association between medically attended infection and subsequent obesity. Parental anxiety and stress could not be assessed but is known to predict care-seeking for viral and other self-limited illnesses (*18*,*19*), albeit inconsistently (*20*). We also did not assess fast food intake and long-term obesity outcomes (*21*). However, the prospective study that initially identified the link between early infection and weight gain had bypassed parental care-seeking by prospective fixed bimonthly assessments by study personnel, and that study prospectively identified upper respiratory infections before 2 years of age, regardless of medical care seeking, as predictive of later overweight as young adults (*8*). Moreover, in a portion of a retrospective study (*13*), clinical infections and obesity outcomes were assessed in discordant same-sex twins. Parental differences in care-seeking were effectively nullified, and the same correlation between infections and later obesity was observed in those twin pairs. Therefore, parental differences in care-seeking behavior probably do not explain the infection–obesity correlation. Our study did not have information on other environmental and social factors such as daycare attendance or presence of older siblings that could be proxy covariates for higher propensity to infection, but those factors have not been shown to predict obesity (*22*,*23*). We were unable to identify other potential covariates that could explain the association between infection and later obesity.

Although the retrospective nature of our study limits our ability to assign causative effect of early infection on later obesity, such an effect is biologically plausible and could reflect an at-risk group or subset of viruses in the infected group. The proportional increase in obesity odds after an increased number of clinical infections reflect the likelihood of exposure to an obesogenic virus. For instance, retrospective human data and prospective animal data have shown a critical correlation of early adenovirus 36 seropositivity and infection with obesity (*24*,*25*), whereas there is no such correlation with other adenoviruses (*26*). In animal models, the effect of this specific adenovirus has been in part through direct action on adipocytes and increased long-term insulin sensitivity (*27*,*28*) promoted by the virus, and those affected might have a healthier type of obesity associated with lower serum lipids and less insulin resistance. A heterogeneous effect on propensity to obesity is consistent with obesity trends in general, and some groups might be more affected by biologic factors such as early infection, similar to social and environmental effects.

In conclusion, our investigation found a correlation between clinical infection in infancy and later weight gain and obesity. The correlation warrants additional study, including prospective study of children stratified by type and severity of infection and of specific viruses such as adenovirus 36, to identify a specific at-risk subgroup or subgroups among children who get frequent early infections. This increased risk for later weight increases is proportional to the number of infections diagnosed and is independent of antimicrobial drug exposure. It is unknown if this observed correlation is causative, if it promotes a relatively healthy obese group, or if it adds to the public health threat of long-term obesity. 
